# Complete Heart Block with Diastolic Heart Failure and Pulmonary Edema Secondary to Enlarging Previously Diagnosed Thrombosed Aneurysm of Sinus of Valsalva in a Patient with History of Autosomal Dominant Polycystic Kidney Disease

**DOI:** 10.1155/2015/281716

**Published:** 2015-03-11

**Authors:** Sherif Ali Eltawansy, Martin Miguel Amor, Maria Joana Thomas, Jeffrey Daniels

**Affiliations:** ^1^Department of Internal Medicine, Monmouth Medical Center, Long Branch, NJ 07740, USA; ^2^Department of Cardiology, Monmouth Medical Center, Long Branch, NJ 07740, USA

## Abstract

Autosomal dominant polycystic kidney disease (ADPKD) is associated with vascular aneurysms that can affect any part of the vascular tree, like ascending aorta or coronary arteries. Sinus of Valsalva is known as an anatomical dilation at the root of aorta above the aortic valve and very few cases show aneurysm at that site in patients with ADPKD. Sinus of Valsalva aneurysm (SVA) can present with rupture and acute heart failure and infective endocarditis or could be asymptomatic accidentally discovered during cardiac catheterization. We report a case of a 76-year-old male with a unique constellation of cardiovascular anomalies associated with ADPKD. Patient was previously diagnosed with aneurysms affecting ascending aorta, sinus of Valsalva, and coronary arteries. Several years later, he came with complete heart block which was discovered later to be secondary to enlargement of his previously diagnosed thrombosed SVA. His case was complicated with acute heart failure and pulmonary edema. *Conclusion*. Patients with ADPKD can present with extrarenal manifestations. In our case, aneurysm at sinus of Valsalva was progressively enlarging and presented with complete heart block.

## 1. Case Presentation

We report a case of a 76-year-old Caucasian male with extensive past cardiac disease history who presented to the hospital with a complete heart block. The patient felt weak and lethargic over the last 2 weeks and it worsened before the admission so he took his blood pressure at home and found his pulse to be very low on the blood pressure machine so he called the ambulance to go to the hospital. There were no symptoms of volume overload like dyspnea on exertion, leg edema, or other symptoms of congestive heart failure (CHF) exacerbation. The patient had a history of a sinus of Valsalva aneurysm (SVA) as well as coronary artery aneurysms; so in the past he had a Bentall procedure, which included placement of a mechanical aortic valve (#23 St. Jude prosthesis) and a conduit replacing the ascending aorta (aortic reconstruction). At that time, there was also a single-vessel bypass using a vein graft to the right coronary artery due to right coronary aneurysm. This was in 1997. He was following up with his cardiologist who did periodic CT/MRI of the chest with contrast to follow the previously diagnosed aneurysms. The repeated chest imaging was showing an ascending thoracic aortic aneurysm as well as the sinus of Valsalva and coronary artery aneurysms and was controlled with medical therapy and follow-up. Warfarin was given for anticoagulation for the metallic aortic valve. The size of thrombosed SVA was gradually increasing over time with 6 cm in diameter in 1999 and then progressed to 7.5 in 2000 and then to 7.3 × 6.5 cm by 2003. By 2004, size went up to 8.5 × 6.4 cm. The size remained stable on the subsequent CT scans that were done regularly. In 2009, the size went up to 8.1 × 7.2 cm. There were multiple cysts throughout the liver, the largest of which were seen in the right hepatic lobe measuring up to 14.7 × 11.3 cm and in the left hepatic lobe measuring 5.1 × 4.1 cm, and these were found unchanged on different CT scans. In 2010 due to uncontrolled hypertension on maximal number of antihypertensive agents, ultrasound of the renal arteries was done to exclude renal artery stenosis and came back negative for any finding. In 2010, patient had an attack of acute back pain and to exclude aortic dissection, CT scan of chest with contrast was done and it showed that the size of thrombosed sinus of Valsalva went up to 9.5 × 8.5 cm but with no actual dissection. MRI of the chest showed larger T1 and T2 hyperintense mass replacing the right lobe of the liver. This demonstrated a new thick wall and demonstrated evidence of hemorrhage within the lesion. This was indicating bleeding into the hepatic and splenic cysts at that time; anticoagulation was stopped till stabilizing the patient. It was found that his INR was 10.1 unexpectedly and could have been secondary to warfarin coagulopathy. INR was reversed with fresh frozen plasma and vitamin K administration. Patient was stabilized at that time and was discharged. 

**Figure 1:**
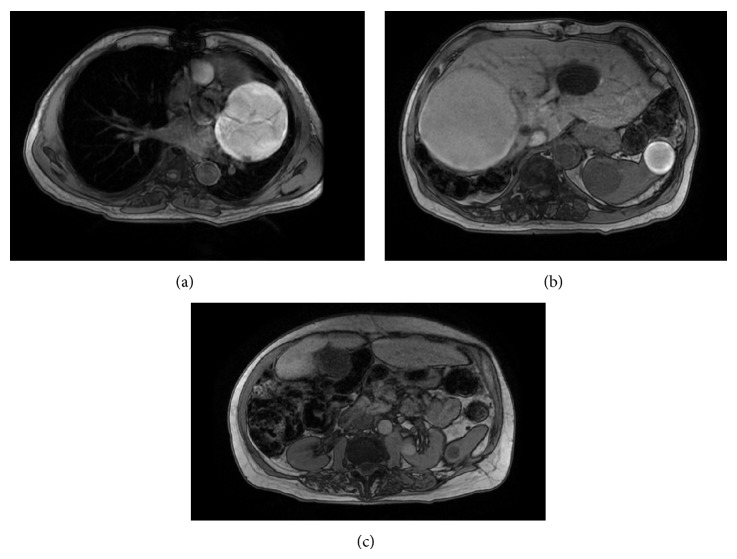
MRI of the abdomen with/without contrast (04-01-2011). (a) A heterogeneous mass is again seen adjacent to the left heart border, measuring approximately 8.5 × 8.0 cm which is partially visualized. Again, a vascular lesion is not excluded. (b) Multiple hepatic cysts are again demonstrated. A large mixed signal mass is again identified in the right lobe of the liver, which in the current study measures 13.0 × 11.7 cm. This measured 19 × 15 cm in the prior study and appears smaller in the current study. Multiple cystic lesions are again noted in the spleen. There is a mass within the spleen anteriorly which again demonstrates a peripheral rim of hypointensity, measuring approximately 3.5 × 3.6 cm. Multiple splenic cysts are noted. (c) Multiple renal cysts are present. There is a new well-circumscribed lesion in the mid pole of the left kidney measuring 1.7 × 1.6 cm which is T1 hyperintense and vaguely T2 hypointense. The subtraction image demonstrated no significant enhancement in this region. This likely represents a hyperdense cyst. There are several T1 hyperintense lesions within the kidneys bilaterally, which are too small to characterize. There are several nonenhancing T2 hyperintensities identified that appear to be within the pancreas; however, these are difficult to evaluate due to overlapping soft tissue structures in this region.

MRI of chest and abdomen with and without contrast was done in April 2011 as a follow-up (Figures 1(a), 1(b), and 1(c)).

In 2013, he was admitted to a hospital for weakness and symptoms of CHF exacerbation and a transthoracic echocardiogram was done showing moderate concentric left ventricular hypertrophy. Left ventricular systolic function is mildly reduced. There is mild global hypokinesis of the left ventricle with ejection fraction 55%. The left atrium was moderately dilated in addition to mechanical aortic valve, moderate mitral regurgitation, prosthetic ascending aorta, a very large and thrombosed SVA, and normal-sized pulmonary artery. Transesophageal echocardiogram was done showing the same findings. This was decided to be managed medically at that time.

Other past medical histories included uncontrolled hypertension with medications that were adjusted to reach the best numbers. He had chronic kidney disease secondary to ADPKD. His serum creatinine level was stable (baseline 157.5 *μ*mol/L) on follow-up and never required dialysis. He had inguinal herniorrhaphy and surgical intervention for left sided hydrocele and spermatocele. He was a nonsmoker and nondrinker. He was living with his family. Family history was negative for premature coronary artery disease or other common cardiac diseases. His father died of a cerebrovascular stroke.

**Figure 2:**
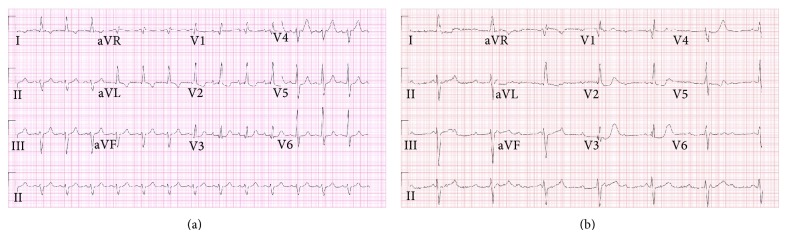
The EKG 4 years before presentation (a) with right bundle branch block with left anterior hemiblock; then progression to complete heart block accompanying the enlargement of thrombosed sinus of Valsalva aneurysm (b).

On admission to our hospital, EKG showed idioventricular rhythm (Figures 2(a) and 2(b)) had replaced the previous sinus rhythm with ventricular rate decreased to 42/minute. Comparing previous EKGs showed that patient had right bundle branch block (RBBB) and left anterior fascicular hemiblock that progressed to complete heart block with AV dissociation and slow ventricular response. Medication list on presentation to us included azilsartan, eplerenone, Tamsulosin, Finasteride, clonidine, warfarin, and furosemide 80 mg tablet daily. He had a reaction to beta-blockers in the form of severe bronchospasm and for that reason he was not on beta-blockers on presentation to us. 

By physical examination, the blood pressure was 144/63 mmHg. Heart rate was 40 beats per minute. Respiratory rate, oxygen saturation on room air, and temperature were normal. Chest was clear on auscultation and cardiac auscultation showed S1 and S2 were normal plus prosthetic mechanical sounds. There was a 2/6 systolic ejection murmur heard over the left mid sternal border. Metallic valve sound was heard. There were no diastolic murmurs or gallops noted. Trivial edema was noted in the legs bilaterally with mild jugular venous distension. Patient was awake and alert with no focal neurological deficit found. 

Chest X-ray on admission showed a large soft tissue mass is again noted consistent with the mass present on the prior MRI that was done 6 months before abutting the left ventricle. Laboratory work showed anemia with HB 106 g/L with low MCV 79.8 fL and MCH 25.5 fL. Troponin was not elevated 0.65. Serum creatinine was 224 *μ*mol/L; BUN was 29.64 mmol/L. Serum potassium was 4.9 mmol/L. This represents worsening of previous kidney chemistry and this was contributed to volume depletion either from poor oral intake or from worsening cardiac functions given his complicated cardiac history.

INR was 5 with PTT 50.8 seconds as he was on warfarin for the prosthetic valve. The plan was to start the patient on dopamine infusion with external pacing pads for the complete heart block. Permanent dual chamber pacemaker was planned but the elevated INR needed to be corrected and fresh frozen plasma units and vitamin K were given. Next day during the stay in the ICU, patient started to develop dyspnea with wheezing and oxygen desaturation and the preliminary diagnosis was congestive heart failure with acute pulmonary edema that could be secondary to diastolic dysfunction exacerbated by plasma transfusion and the new onset complete heart block. Furosemide was given intravenously. Blood pressure was 163/70 with heart rate 53 while he was still on dopamine infusion. The patient was sent to the operation room immediately to place the permanent pacemaker and this was done under general anesthesia with endotracheal intubation (ETT). The patient returned back to the ICU on a ventilator and weaning was not possible postoperatively due to oxygen desaturation and altered mental status. Patient was put on propofol and fentanyl infusions. The repeated daily chest X-rays were showing cardiomegaly with hazy lung opacities at the base concerning pleural effusion and a mediastinal mass on left hemithorax representing the previously recognized thrombosed SVA.

**Figure 3:**
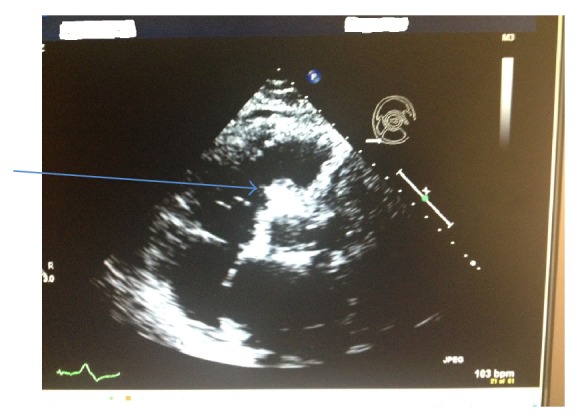
2D echocardiogram shows the large thrombosed sinus of Valsalva aneurysm (blue arrow). Report: Echocardiogram was done showing no change in ejection fraction from previous studies (55%) with left ventricle concentric hypertrophy in addition to the prosthetic aortic valve, dilated left atrium, a very large and thrombosed sinus of Valsalva aneurysm, mildly dilated inferior vena cava, a pacemaker lead in the right ventricle, and a catheter/pacemaker lead seen in the RA cavity. It was noted that the right ventricle systolic pressure was elevated at 40–50 mmHg.

Echocardiogram was done showing a very large and thrombosed sinus of Valsalva aneurysm (Figure 3). Trials of weaning from ventilator failed and patient seemed to still have persistent pulmonary vascular congestion that required continuous diuresis. A desperate trial was made by ordering Lyme disease antibody titer as a possible reason of the previously diagnosed heart block and it came back negative.

**Figure 4:**
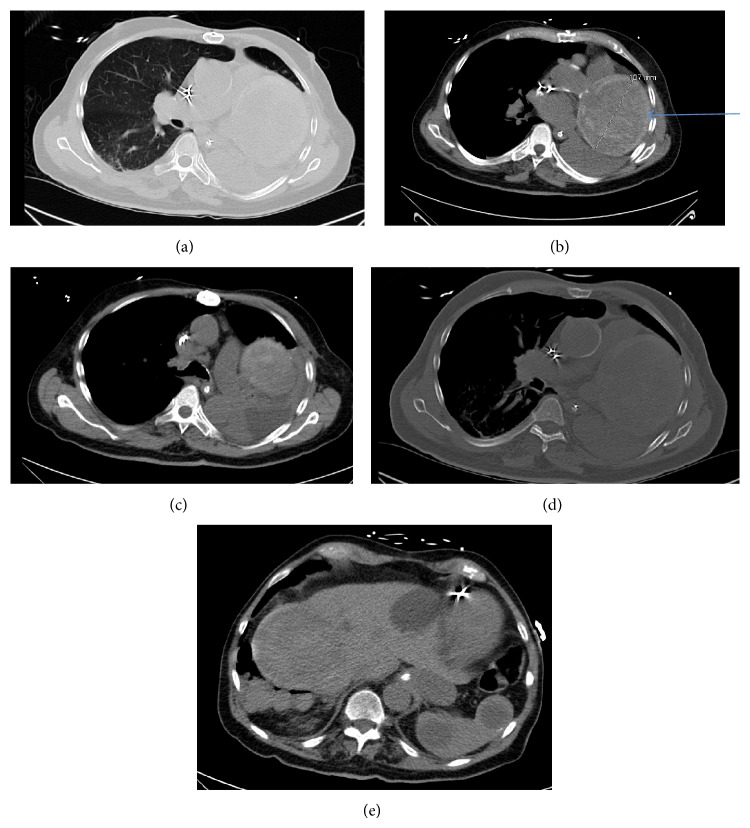
CT of the chest without contrast (March 19, 2014). (a) Left lung volume loss was noted in the left lung with underlying atelectatic change. A moderately sized pleural effusion was noted, which contained a second component measuring above that of simple fluid attenuation. These findings might represent an element of a hemorrhagic component. It was possible that this was atelectatic lung adjacent to the pleural effusion. An endotracheal tube was seen with the tip in the distal trachea near the carina. Low attenuation material was present within the left main stem bronchus, causing complete occlusion. Atelectatic changes were present in the right lung base with an early consolidation seen. Numerous nodules are seen throughout the lung fields. Scattered small blebs are noted in the right lung. (b) Heart was showing a heterogeneous mass (blue arrow), again noted to be associated with the left atrium and likely arising from the aortic root which measured 9.5 × 11.1 cm slightly larger in comparison to the MRA of the chest dated June 10, 2008; at that time it measured 8 cm in maximum diameter. There was a mediastinal shift to the left. (c) A calcified right hilar lymph node was noted. A prominent pretracheal lymph node measured 2 × 1.5 cm and could be a combination of 2 adjacent nodes. This was relatively stable. Another adjacent lymph node was measuring 9.7 cm slightly larger. A few other enlarged lymph nodes in the prevascular space were also present. Examination was difficult due to lack of intravenous contrast. (d) Vascular: atherosclerotic calcifications were seen in the aorta and its branches. There was a stable aneurysm of the thoracic aorta measuring 4.4 cm. (e) An enteric tube was seen coursing into the stomach with the tip off the field of view. Multiple splenic cysts were again noted, grossly stable in appearance. One of these cysts contains a calcification. Multiple hepatic cysts were again noted. A small hiatal hernia was seen. A heterogeneous complex cystic mass was again noted in the right hepatic lobe, which measured 9 × 8.3 cm, grossly stable in appearance compared to the most recent MRI from 2012. Multiple renal cysts were seen. Pancreatic calcifications were noted.

BNP (B-natriuretic peptide level) was 3419 ng/L. Heparin infusion was started as patient needed anticoagulation for the mechanical aortic valve. Tube feeding was started via the orogastric tube (OGT). Eplerenone tablet was resumed via the OGT in an effort to help improve the worsening cardiac function. The daily diuresis with resultant negative fluid balance improved pulmonary vascular congestion and right-sided pleural effusion but there was a persistent opacification of the left hemithorax. BNP level was going down. There was a mass-like opacity on the left hemithorax which was interpreted with the preliminary differential diagnosis like pleural effusion or pneumonic consolidation. The furosemide intake improved the pleural effusion on daily chest X-rays but in the meanwhile blood pressure was dropping from overdiuresis and kidney functions started to worsen from the fluid shift away from the kidneys. So the diuresis was stopped. Hypernatremia (NA 157 mmol/L) developed due to free water loss and free water was given via the orogastric tube. Amlodipine, furosemide, and eplerenone were held given the low blood pressure and worsening kidney functions. Intravenous fluids were tried very cautiously due the complicated heart failure and hypervolemia. The goal was to keep INR between 2 and 3 and warfarin was stopped when INR was over that goal; then patient was kept on heparin infusion only given the worsening left-sided pleural effusion with the fear of developing hemorrhage into it. There was cream-colored moderate-to-large amount of secretions which was suctioned from his endotracheal tube. Sputum culture came back with* Klebsiella pneumonia*; so pneumonia was thought to be present and vancomycin plus ceftriaxone that was shifted to tazobactam-piperacillin was started intravenously. Vancomycin then was stopped. Then antibiotic was narrowed down to ceftriaxone according to the sensitivity result of the sputum culture. The decision was to do CT scan of the chest but without contrast due to worsening kidney functions. The CT scan of the chest showed moderate left-sided pleural effusion plus enlargement of the previously recognized thrombosed SVA (Figures 4(a), 4(b), 4(c), 4(d), and 4(e)). This leads to the idea that heart block happened secondary to enlarging SVA. Tapping of left-sided pleural effusion came back with 100 cc that was nonhemorrhagic. The analysis came back exudative and was thought to be secondary to a possible pneumonia on the same side of the lung. It was negative for malignant cells.

Leukocytosis was getting worse and WBC went up to 26 × 10^9^/L. Repeat sputum culture was sent again and showed* Pseudomonas aeruginosa*. Antibiotic was changed from ceftriaxone to ertapenem to cefepime according to the sensitivity result. Still there was no fever and actually leukocytosis improved after the change in antibiotics and went down to 12.9 × 10^9^/L. Patient developed diarrhea and stool cultures came back positive for* Pseudomonas aeruginosa*. Kidney functions were worsening and serum creatinine went up to 267.75 *μ*mol/L and BUN went up to 44.28 mmol/L. Kidney functions started to improve after gentle hydration and free water intake via orogastric tube but again pleural effusion on the left side was persistent on daily chest X-rays and BNP level was high so furosemide was given cautiously.

The cardiologist told the family that patient might need to be transferred to a specialized hospital where he will have a surgical repair of the enlarged thrombosed SVA. The prognosis was poor and weaning from mechanical ventilator failed and patient stayed on it for 15 days with no improvement. After discussion among family members and given the poor prognosis, the plan was cancelled and it was decided to do a terminal weaning and refer the patient to hospice.

**Figure 5:**
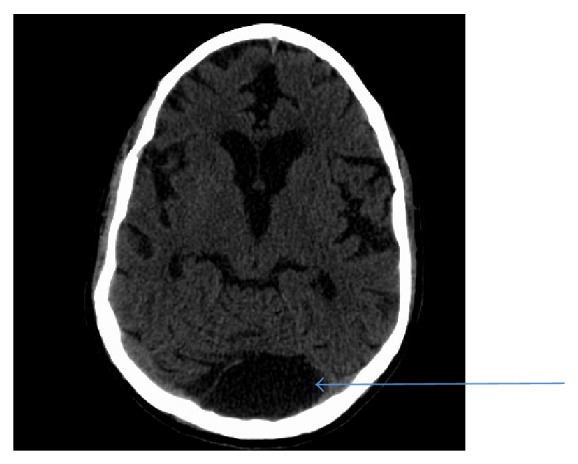
CT of the head without contrast. The ventricles and sulci are prominent, most consistent with global cerebral volume loss. Hypoattenuation of the periventricular white matter is most consistent with chronic small vessel ischemic disease. There is no evidence for intraparenchymal mass lesion or hemorrhage. A large CSF-density space (blue line) is seen posterior to the midline cerebellum, measuring 5.0 × 2.9 cm in image 29 of series 2, most consistent with arachnoid cyst. The gray-white junction is intact. Mild polypoid mucosal thickening is seen within the left maxillary sinus. Mild mucosal thickening is also seen within the right sphenoid sinus. A large right mastoid effusion is seen.

After terminal weaning, the patient was still surviving off the ventilator and oxygen saturation was stable on nasal cannula. His eyes were opened and he was noncommunicating and the differential diagnosis was a cerebrovascular stroke. So the patient was sent for a CT scan of the head and came back with a large retrocerebellar arachnoid cyst (Figure 5). The confusion and unresponsiveness were then attributed to anoxic encephalopathy. The patient stayed for 2 days in the hospice ward and then died after his family had clarified that they do not wish to do a resuscitation to him.

## 2. Discussion

Autosomal dominant polycystic kidney disease (ADPKD) is the result of three defective genes on chromosomes 4 and 16. ADPKD is a genetically heterogeneous disease identified by two phenotypically similar forms associated with several mutations in those two genes. Mutations of PKD1 gene, encoding the polycystin-1 protein, result in ADPKD type I (ADPKD1) which is responsible for approximately 85% of ADPKD cases. Gene PKD2 mutations, encoding the polycystin-2 protein, result in ADPKD type II (ADPKD2) corresponding to 15% of ADPKD cases [1].

The precise processes leading to cyst formation and loss of renal function remain incompletely understood. Several mechanisms contributing to the cyst formation have been identified, including an imbalance between epithelial cell proliferation and apoptosis, secretor defects, altered cell-matrix interactions, cell polarity, ciliary dysfunction, and altered intracellular signaling [2].

Diagnostic criteria of ADPKD depend on the age; among individuals between 15 and 39 years of age, it is expected to find at least three unilateral or bilateral kidney cysts on ultrasound. Among individuals of 40 to 59 years of age, it is expected to find at least two cysts in each kidney. Among individuals of 60 years or older, at least four cysts in each kidney are expected for diagnosis [3]. Cysts may also be seen in the liver and pancreas. Hepatic cysts, for example, can be detected in over half of cases and are more commonly seen in women and in patients over the age of 40 years [4].

Extrarenal features of autosomal dominant polycystic kidney disease (ADPKD) are characterized by cysts in the kidneys and, in many cases, are associated with cysts in the liver and pancreas that can be helpful in confirming the diagnosis. In addition, patients may have a variety of other abnormalities, many of which are consistent with a generalized defect in epithelial cell differentiation and/or extracellular matrix function as a primary expression of the genetic abnormality in this disorder [5]. The major extrarenal complications of ADPKD are as follows:

- cerebral aneurysms,
- hepatic and pancreatic cysts,
- cardiac valve disease,
- colonic diverticula,
- abdominal wall and inguinal hernia,
- seminal vesicle cysts [6, 7].

Our reported case had multiple hepatic, renal, pancreatic, and splenic cysts in addition to history of inguinal hernia, which lead us to a full blown picture of ADPKD.

Our patient had an arachnoid cyst accidentally on CT brain that was done in the late part of his hospital course as the indication was unresponsiveness and confusion that turned out to be secondary to anoxic brain injury and long term ventilation on sedatives. Arachnoid cyst here was asymptomatic. A study showed that among 247 patients with ADPKD who underwent magnetic resonance imaging or high-resolution contrast enhanced computerized tomography, intracranial arachnoid cysts were found in 8.1% with ADPKD compared to 0.8% in a control group without ADPKD matched for age, sex, and method of imaging. Multiple intracranial arachnoid cysts were found in two patients. Polycystic liver disease was present in 85.0% of the patients with intracranial arachnoid cysts compared to 52.4% of the patients without. Pineal cysts were found in 0.8% and choroid plexus cysts were found in 1.2% but this was not different from the control population. None of the intracranial cysts was symptomatic and none was treated surgically [8].

Cardiovascular malformations of ADPKD were remarkably found in our reported case. Malformations of selected vasculature, including intracranial aneurysms and aortic root dilatation (normal diameter ≤35 mm), may be due to altered expression and/or function of the PKD gene in arterial smooth muscle cells [9]. The most common abnormalities in cases with ADPKD include mild mitral valve prolapse and aortic regurgitation; less frequent lesions include mitral and/or tricuspid regurgitation. Generalized abnormalities in collagen and/or extracellular matrix may be responsible for the valve disease in ADPKD; aortic regurgitation, for example, may result from dilatation of the aortic root and annulus [10, 11].

Preliminary evidence suggests that PKD may also be associated with an increased incidence of coronary aneurysms (defined as an increased diameter of ≥50 percent or pathologic ectasia) and coronary artery dissection. Coronary artery dissection has been described in isolated case reports [12]. Asymptomatic pericardial effusions appear to occur at increased frequency in patients with ADPKD [7]. Our reported case had pericardial effusion around 1997 with missing details as it was a long time ago in another hospital.

ADPKD is considered as cardiorenal syndrome (CRS) type 4:

- acute cardiorenal syndrome CRS type 1: abrupt worsening of cardiac function leading to acute kidney injury (AKI),
- chronic cardiorenal syndrome CRS type 2: chronic abnormalities in cardiac function causing progressive chronic kidney disease (CKD),
- acute renocardiac syndrome CRS type 3: sudden worsening of renal function causing acute cardiac dysfunction,
- chronic renocardiac syndrome CRS type 4: condition of primary CKD leading to an impairment of the cardiac function and/or increased risk of adverse cardiovascular events,
- secondary cardiorenal syndrome CRS type 5: systemic disorders (e.g., sepsis) causing both cardiac and renal dysfunction [13].
- Therefore, the cardiovascular complications seen in ADPKD patients begin to be recognized not only as a consequence of declining kidney function, but also as a defect due to the loss of polycystin-1 and/or polycystin-2 function in cardiovascular organs [14].

Polycystin may direct the characteristics of the bond between type 4 collagen-anchoring fibrils and the proteoglycans of the lamina densa in the aortic valve cusps. Therefore, if this bond or composition of the basal lamina is flawed, a possible defect in autosomal dominant polycystic kidney disease, the result would be a gross structural defect: loss of continuity between the aortic valve leaflet endothelium and the cardiac skeleton. Therefore, the association between ADPKD and acquired SVAs may be the production of sinus of Valsalva tissue with atypical composition that after repeated stress becomes fibrotic and dilates. Thus, these aneurysms are not true aneurysms. Rather, their walls are composed of avulsed fibrous tissue [15, 16]. Sinus of Valsalva aneurysms are rare and are more common in men [17]. The right sinus is involved in most cases, the noncoronary sinus in up to 15%, and the left sinus only seldom [18]. It is considered as a rare congenital anomaly caused by a defect of continuity between the aortic media and the annulus fibrosis. SVA was first described by Hope in 1837. Rare association with polycystic kidney has also been reported [18, 19]. Secondary types of SVAs occur following aortic root fibrosis due to endocarditis; syphilis; iatrogenic trauma (postoperative ventricular septal defect and valve repairs); degenerative states such as atherosclerosis; or conditions associated with altered extracellular matrix such as cystic medial necrosis, Marfan's syndrome, aortoannular ectasia, osteogenesis imperfecta, pregnancy, Behcet's disease, and congenital types [16]. SVA is reported to represent less than 0.5% of all lesions requiring cardiopulmonary bypass for repair [20] and most acquired SVAs will not be diagnosed until rupture, except in patients followed up for bacterial endocarditis, conduction disorders, or connective tissue disease. Once ruptured, whether the onset of symptoms is gradual or acute will depend not only on the shunt flow and associated cardiac lesions, but also on the patient's systemic condition. Several techniques of repairing a ruptured SVA have been reported and their relative efficacies depend on the location of the aneurysm and the presence of accompanying cardiac lesions [21, 22].

Our reported case had a new onset complete heart block that necessitated a permanent pacemaker placement. The finding of enlarging previously known thrombosed SVA that was already operated on before with an aortic reconstruction had guided us to the assumption that the heart block occurred secondary to the complicated progressive thrombosed SVA.

The pattern of presentation of SVA does vary widely and unruptured aneurysms are often “silent” or present only with vague nonspecific symptoms. Rare manifestations include cerebrovascular accidents, myocardial ischemia, mitral incompetence, right ventricular outflow tract obstruction, left ventricular outflow tract obstruction, and atrial fibrillation. Cardiac conduction disturbance due to SVA can occur at several levels, including sinoatrial conduction disruption and various levels of his bundle block. Transient atrioventricular block and persistent complete atrioventricular block are also reported [23]. Theories to explain previously reported cases were made and agreement was assumed that either direct pressure from the expanding aneurysm or low-grade inflammation can lead to AV conduction defects [24]. One of the reported cases had a complete heart block secondary to SVA. The sinus rhythm was restored after surgical decompression of the interventricular septum. This suggests that complications such as heart block can be prevented by operating as soon as possible on an unruptured SVA that has eroded into the interventricular septum [25].

Another case was reported with SVA presenting as complete heart block. The patient presented unruptured aneurysm that had extended into the interventricular septum and, presumably through compression, had compromised normal AV nodal/His bundle function and resulted in complete heart block. Case reports of the latter association are very rare. Therefore, sinus of Valsalva aneurysm deserves to be considered as a rare cause of complete heart block [26, 27].

There were 7 reported cases that had SVA with intraseptal extension. There were conduction abnormalities and four patients had complete heart block. The mechanism for atrioventricular block is easily explained by extension of the SVA into the vicinity of the conduction tissue [27]. In our reported case, the progression of the size of SVA had led to progression from bifascicular block (right bundle branch block and left anterior hemiblock) to complete heart block.

## 3. Conclusion

To summarize, our case represents a unique constellation of extrarenal manifestations of ADPKD that included coronary artery aneurysm, ascending aorta aneurysm, sinus of Valsalva aneurysm with thrombosis and progression, pericardial effusion, hepatic, renal, splenic, and pancreatic cysts, arachnoid cyst and spermatic cyst, and inguinal hernia. His presentation to our hospital with complete heart block which could be secondary to enlarging thrombosed sinus of Valsalva aneurysm increases the uniqueness of our reported case to show a good example of a disease affecting renal and cardiovascular systems secondary to an inherited disease. To our knowledge, it is the first case that is known to have ADPKD to present with complete heart block secondary to progression of thrombosed SVA. Patient had a full blown picture of ADPKD and died of advanced cardiovascular complications.

## Learning Objective

Autosomal dominant polycystic kidney disease can present with extrarenal cardiovascular manifestation. Vascular aneurysms can affect any part of the vascular tree including the sinus of Valsalva. When affected, it could present with different manifestations or it could be asymptomatic. Presenting with complete heart block is uncommon and has to be expected in the same time.
